# Single-cell RNA sequencing of human femoral head **in vivo**

**DOI:** 10.18632/aging.203124

**Published:** 2021-06-10

**Authors:** Xiang Qiu, Ying Liu, Hui Shen, Zun Wang, Yun Gong, Junxiao Yang, Xiaohua Li, Huixi Zhang, Yu Chen, Cui Zhou, Wanqiang Lv, Liang Cheng, Yihe Hu, Boyang Li, Wendi Shen, Xuezhen Zhu, Li-Jun Tan, Hong-Mei Xiao, Hong-Wen Deng

**Affiliations:** 1 Center for System Biology, Data Sciences, and Reproductive Health, School of Basic Medical Science, Central South University, Yuelu, Changsha 410013, China; 2 Tulane Center of Biomedical Informatics and Genomics, Deming Department of Medicine, School of Medicine, Tulane University, New Orleans, LA 70112, USA; 3 Xiangya Nursing School, Central South University, Changsha 410013, China; 4 Laboratory of Molecular and Statistical Genetics, College of Life Sciences, Human Normal University, Changsha 410081, China; 5 Department of Orthopedics, Xiangya Hospital, Central South University, Changsha 410008, China; 6 Department of Orthopedics and National Clinical Research Center for Geriatric Disorders, Xiangya Hospital, Central South University, Changsha 410008, China

**Keywords:** single-cell RNA sequencing, bone cell, immune cell, bone metabolism, cell-cell communication

## Abstract

The homeostasis of bone metabolism depends on the coupling and precise regulation of various types of cells in bone tissue. However, the communication and interaction between bone tissue cells at the single-cell level remains poorly understood. Thus, we performed single-cell RNA sequencing (scRNA-seq) on the primary human femoral head tissue cells (FHTCs). Nine cell types were identified in 26,574 primary human FHTCs, including granulocytes, T cells, monocytes, B cells, red blood cells, osteoblastic lineage cells, endothelial cells, endothelial progenitor cells (EPCs) and plasmacytoid dendritic cells. We identified *serine protease 23* (*PRSS23*) and *matrix remodeling associated protein 8* (*MXRA8*) as novel bone metabolism-related genes. Additionally, we found that several subtypes of monocytes, T cells and B cells were related to bone metabolism. Cell-cell communication analysis showed that collagen, chemokine, transforming growth factor and their ligands have significant roles in the crosstalks between FHTCs. In particular, EPCs communicated with osteoblastic lineage cells closely via the "COL2A1-ITGB1" interaction pair. Collectively, this study provided an initial characterization of the cellular composition of the human FHTCs and the complex crosstalks between them at the single-cell level. It is a unique starting resource for in-depth insights into bone metabolism.

## INTRODUCTION

Compared with other tissues in the body, bone is a relatively dynamic organ, which undergoes significant turnover during life [1]. The coupling and precise regulation between bone cells affect the homeostasis of bone metabolism, including bone formation by osteoblast, bone resorption by osteoclast and regulation by osteocyte [2, 3]. In addition, bone microenvironment is a complex system containing various other types of cells, such as stromal cell, immune cells, endothelial cells, which also influence bone metabolism via complex crosstalks [4]. For instance, monocytes can regulate bone remodeling by secretion of various cytokines, such as bone morphogenetic protein 2 (BMP2)^,^ which in turn promote the osteogenic differentiation by mesenchymal stem/stromal cells [5]. Resting T cells have a protective role of bone [6], while activated T cells increase the production of receptor activator of NF-kappaB ligand (RANKL) and tumor necrosis factor alpha (TNF-α) to promote osteoclast formation and subsequent bone loss under inflammatory conditions [7]. B cells can regulate osteoclastogenesis by expressing osteoclast differentiation factor (ODF)/RANKL [8]. However, current strategies for bone study are based on whole cell population of bone by bulk sequencing of al the cells for bone tissue [9, 10]. The approach ignores the heterogeneity between individual cells and lack the accuracy and resolution to characterize regulation and crosstalks between bone tissue cells.

Single-cell RNA sequencing (scRNA-seq) provides an opportunity to explore the heterogeneity of complex tissues and cell-to-cell interactions at high resolution [11, 12]. Although flow cytometry is a prominent technique for categorizing cells, which can identify the single cell through the expression of both cell surface and (or) intracellular proteins, it has been limited to probing a few selected proteins [13, 14]. Similarly, magnetic activated cell sorting (MACS) and immunohistochemistry (IHC) also have this limitation. And *in situ* hybridization (ISH) has been limited to probing a few selected RNAs. These single-cell approaches can only focus on information of the selected RNAs or proteins [13], while scRNA-seq can provide a broad characterization of the transcriptome profile. Besides, compared with the traditional bulk-RNA sequencing, scRNA-seq provides information of cellular biology at higher resolution and with more accuracy [15]. scRNA-seq has been successfully applied to reveal the transcriptional diversity of murine bone marrow-derived mesenchymal stem cells (BM-MSCs) [16], and to identify differential expression genes (DEGs) between human Wharton's jelly stem cells and human BM-MSCs [17]. However, the cellular composition of bone tissue cells and the crosstalks between them at single-cell resolution remains unknown.

Here, we applied scRNA-seq technology to characterize cellular heterogeneity at single-cell level in freshly isolated bone tissue cells from human femoral head. We identified *serine protease 23* (*PRSS23*) and *matrix remodeling associated protein 8* (*MXRA8*) as novel bone metabolism-related genes. Moreover, we defined distinct subtypes of monocytes, T and B cells in bone microenvironment. We further discussed their relationship with bone metabolism and re-constructed the communication networks of cells in human femoral head. We believe that the global single-cell profile of how different types of human femoral head tissue cells work together would promote our comprehensive understanding of bone metabolism, and provide some novel insights into the prevention and treatment of skeletal diseases, such as osteoporosis and osteoarthritis.

## RESULTS

### scRNA-seq analysis reveals distinct cell types in human femoral head

**Figure 1:**
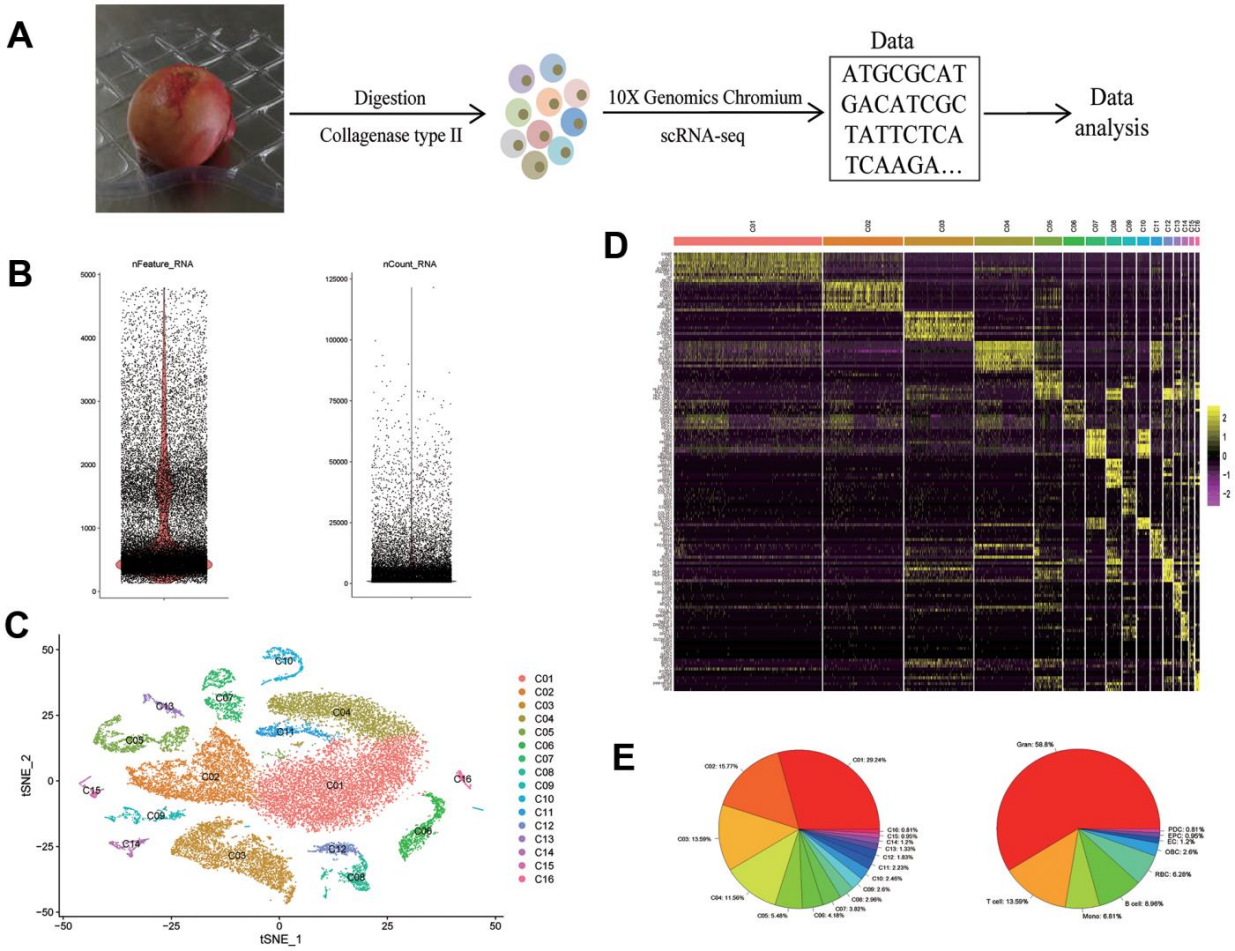
**scRNA-seq reveals the cell populations of the human femoral head.** (**A**) Study overview. (**B**) After QC, the number of genes (left) and RNA molecules (right). (**C**) t-SNE plot shows the color-coded clustering of human femoral head tissue cells. (**D**) Heat map shows the top 10 genes with the highest avg_logFC of each cluster. (**E**) The proportion of each cluster (left) and each cell type (right). scRNA-seq: single-cell RNA sequencing: Gran: granulocyte; Mono: monocyte; RBC: red blood cell; OBC: osteoblastic lineage cell; EC: endothelial cell; EPC: endothelial progenitor cell; PDC: plasmacytoid dendritic cell.

We performed scRNA-seq analyses on femoral head tissue cells from four human subjects (Figure 1A). The gene expression profiles between samples have a strong correlation, suggesting that there is no obvious batch effect between samples (*R* > 0.96; Supplementary Figure 1). After merging of the four datasets and QC, we obtained a cell-gene matrix of 26,574 cells, with an average of 1035 genes detected per cell (Figure 1B). Then we clustered cells into 16 distinct clusters (Figure 1C), and identified the cluster-specific markers (Figure 1D).

Among these clusters, we identified that, 1) clusters C01, C02, C04, C11 were *CD11b*^+^*CD66b*^+^ granulocytes; 2) cluster C03 was CD3^+^ T cells; 3) clusters C05 and C13 were CD14^+^ monocytes; 4) clusters C06, C08, C12 were CD19^+^CD79A^+^*CD20*^+^ B cells; 5) clusters C07 and C10 were *CD235a*^+^ red blood cells (RBCs); 6) cluster C09 was osteoblastic lineage cells; 7) cluster C14 was *CD31*^+^*VWF*^+^ endothelial cells (ECs); 8) cluster C15 was *CD117*^+^*CD133*^+^ endothelial progenitor cells (EPCs); 9) cluster C16 was *GZMB*^+^*IL3RA*^+^ plasmacytoid dendritic cells (PDCs). Proportions of each cluster and each cell type were shown in Figure 1E, respectively.

### Functional analyses and hub genes identification for DEGs of osteoblastic lineage cells

The osteoblastic lineage cells were a complex cell population which contained BM-MSCs, osteoblasts, osteocytes and chondrocytes, and we showed the expression of cell-specific markers by the violin plot (Supplementary Figure 2A).

To further study the biological functions of osteoblastic lineage cells, we performed GO and KEGG enrichment analyses based on the DEGs of osteoblastic lineage cells (Supplementary Tables 2, 3). GO enrichment analysis identified abundant terms related to bone metabolism, such as “extracellular structure organization”, “extracellular matrix organization”, “establishment of protein localization to organelle”, “skeletal system development”, and “ossification” (Supplementary Figure 2B). Several signal pathways related to bone metabolism were revealed by KEGG enrichment analysis (Supplementary Figure 2B), such as “PI3K-Akt signaling pathway”, “Rap1 signaling pathway”, and “TGF-beta signaling pathway”.

**Figure 2:**
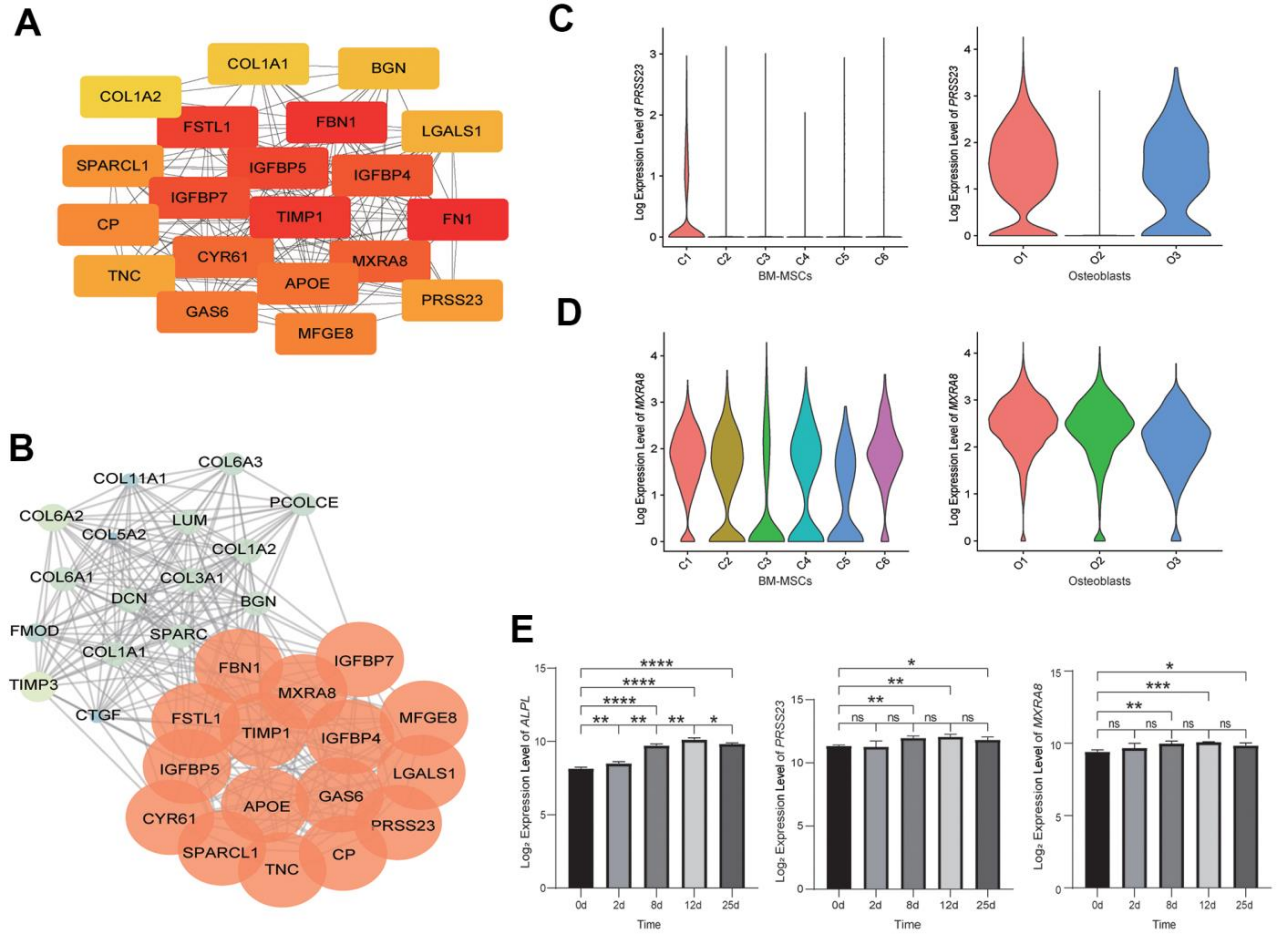
**Analysis of osteoblastic lineage cells.** (**A**) Gene network analysis of DEGs. The top 20 hub genes in the network. The color changes from yellow to red, indicating low to high connectivity. (**B**) The top MCODE-score module (module 1) screened from the PPI network. The color changes from blue to red, indicating low to high MCODE-score. (**C**) The expression level of *PRSS23* in BM-MSCs (left) and osteoblasts (right). C1: osteoblast precursor; C2: adipocyte precursor; C3: terminal 1; C4: terminal 2; C5: contaminated; C6: chondrocyte precursor; O1: pre-osteoblast (early osteoblast); O2: mature osteoblast; O3: undetermined osteoblast (early osteoblast). (**D**) The expression level of *MXRA8* in BM-MSCs (left) and osteoblasts (right). (**E**) The expression levels of *alkaline phosphatase* (*ALPL*), *PRSS23* and *MXRA8* during *in vitro* osteogenic differentiation from BM-MSCs (left to right). X-axis represents time (days) of induce differentiation and y-axis reflects log_2_-normalized gene expression levels. Stars indicate significance level of gene expression difference between two samples by t-test. ns, not significant; *, *p* value < 0.05; **, *p* value < 0.01; ***, *p* value < 0.001; ****, *p* value < 0.0001.

To identify the hub genes, which are genes with a high degree of connectivity, in the DEGs of osteoblastic lineage cells, a PPI network of DEGs was constructed (Supplementary Figure 2C), and the top 20 hub genes with a high degree of connectivity were detected (Figure 2A). These top 20 hub genes were enriched in the process related to bone metabolism (Supplementary Table 4 and Supplementary Figure 2D), such as “extracellular structure organization”, “extracellular matrix organization”, “ossification”, “skeletal system development” and “osteoblast differentiation”. We also detect seven significant modules in the PPI network (Figure 2B and Supplementary Figure 2E, Supplementary Table 5). We used genes in the most significant module, module 1 (score = 19.097, with 32 nodes and 296 edges) for a GO enrichment analysis (Supplementary Figure 2D and Supplementary Table 6), and found that genes in module 1 were significantly related to extracellular structure, extracellular matrix, collagen fibril, ossification, skeletal system, etc. The biological process analysis of the top 16 genes in module 1 was shown in Supplementary Figure 2F.

Among the hub genes in the DEGs network and the hub genes in the most significant module (module 1) of the PPI network, we found that most of these genes were known to be related to bone metabolism (Supplementary Table 7). However, two genes, *PRSS23* and *MXRA8*, were rarely reported to regulate bone metabolism. In addition, *PRSS23* was highly expressed in osteoblast precursors and early osteoblasts (pre-osteoblast and undetermined osteoblast) (Figure 2C), and *MXRA8* was highly expressed in both BM-MSCs and osteoblasts (Figure 2D). During the process of osteogenic differentiation by BM-MSCs *in vitro*, the expression levels of *PRSS23* and *MXRA8* were significantly increased (Figure 2E). Therefore, we speculate that *PRSS23* and *MXRA8* may play important roles in bone metabolism.

### scRNA-seq analysis reveals distinct subtypes in monocytes, T cells and B cells in human femoral head

To study the cellular heterogeneity of monocytes, T cells and B cells in bone tissue, we extracted 1,810 CD14^+^ monocytes, 3,612 CD3^+^ T cells and 2,382 CD19^+^CD79^+^*CD20*^+^ B cells from the original dataset for further analyses.

**Figure 3:**
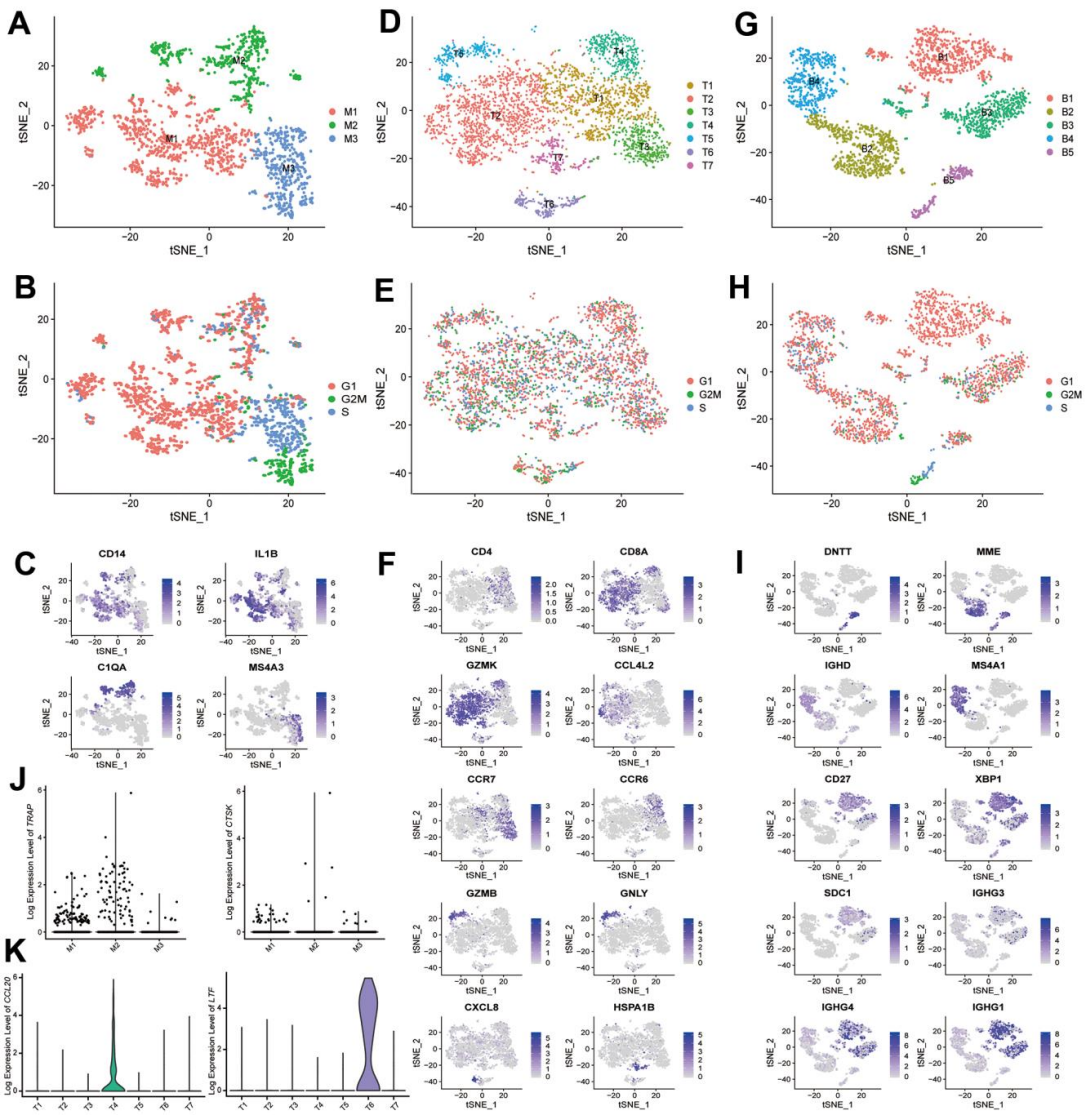
**scRNA-seq analysis reveals different cell subtypes in monocytes, T cells and B cells.** (**A**) t-SNE plot shows the color-coded clustering for monocytes. Monocytes: M1-M3. (**B**) t-SNE plot shows the cell cycle status of monocytes. (**C**) Monocyte subtypes signature genes, embedded on t-SNE dimension reduction map, and colored by gene expression levels. (**D**) t-SNE plot shows the color-coded clustering for T cells. T cells: T1-T7. (**E**) t-SNE plot shows the cell cycle status of T cells. (**F**) T cell subtypes signature genes, embedded on t-SNE dimension reduction map, and colored by gene expression levels. (**G**) t-SNE plot shows the color-coded clustering for B cells. B cells: B1-B5. (**H**) t-SNE plot shows the cell cycle status of B cells. (**I**) B cell subtypes signature genes, embedded on t-SNE dimension reduction map, and colored by gene expression levels. (**J**) The expression level of *TRAP* (left) and *CTSK* (right) in monocytes subtypes. (**K**) The expression level of *CCL20* (left) and *LTF* (right) in T cells subtypes.

Among the monocytes (Figure 3A–3C), we identified three putative subtypes: *IL1B*^+^ monocytes (M1), *C1QA*^+^ monocytes (M2), and *MS4A3*^+^ granulocyte-monocyte progenitors (M3). In the T cells (Supplementary Figure 4D–4F), we identified one CD4 cluster (T1), and six CD8 clusters: *GZMK*^+^*CCL4L2*^+^ T cells (T2), *CCR7*^+^ T cells (T3), *GZMK*^+^*CCR6*^+^ T cells (T4), *GZMB*^+^*GNLY*^+^ T cells (T5), *GZMK*^+^*CXCL8*^+^ T cells (T6), and *GZMK*^+^*HSPA1A*^+^ T cells (T7). Within the B cells (Figure 3G–3I), we identified *DNTT*^+^/*MME*^+^ pre-B cell (B2 and B5), *MSHA1*^+^ mature/activated B cell (B4), CD27^+^ memory B cell (B3), and a plasmablast cluster (B1) with high expression of immunoglobulin genes and *XBP1* (a transcription factor for plasma cell differentiation) [18].

**Figure 4:**
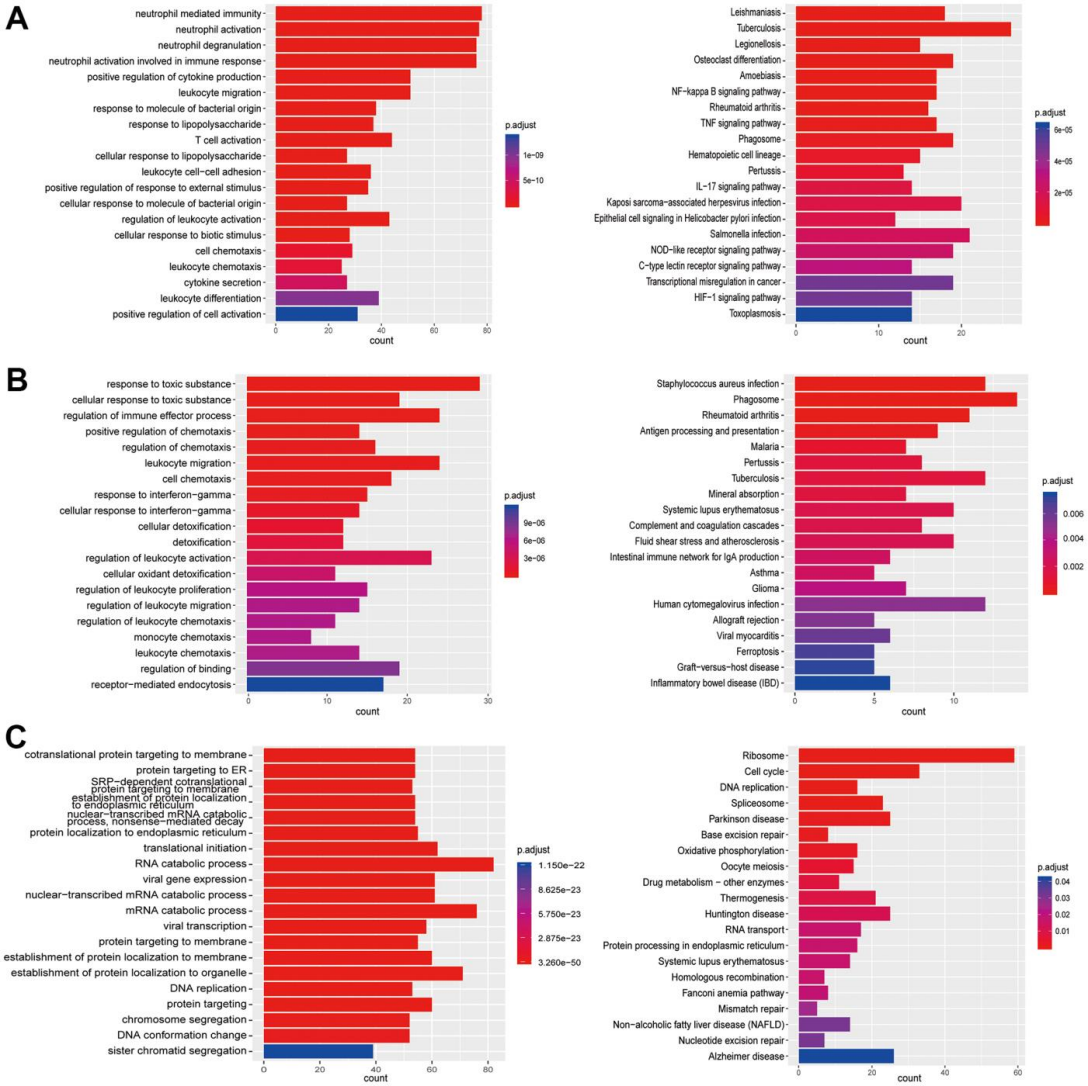
**Enrichment analysis of subtypes in monocytes (M1-M3).** (**A**) GO (left) and KEGG (right) enrichment analysis of DEGs in M1. (**B**) GO (left) and KEGG (right) enrichment analysis of DEGs in M2. (**C**) GO (left) and KEGG (right) enrichment analysis of DEGs in M3.

**Figure 5:**
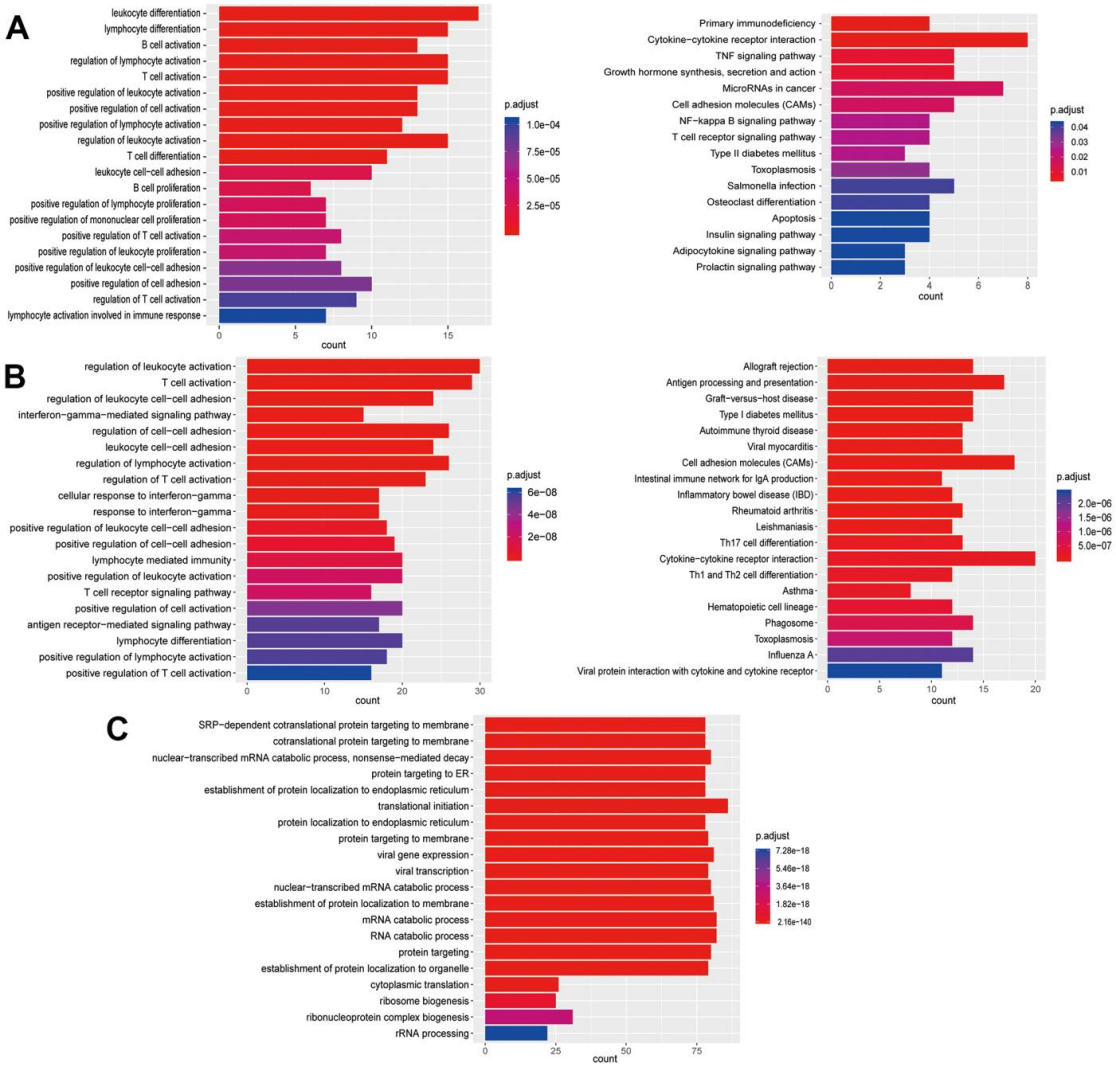
**Enrichment analysis of subtypes in T cells (T1-T3).** (**A**) GO (left) and KEGG (right) enrichment analysis of DEGs in T1. (**B**) GO (left) and KEGG (right) enrichment analysis of DEGs in T2. (**C**) GO enrichment analysis of DEGs in T3.

**Figure 6:**
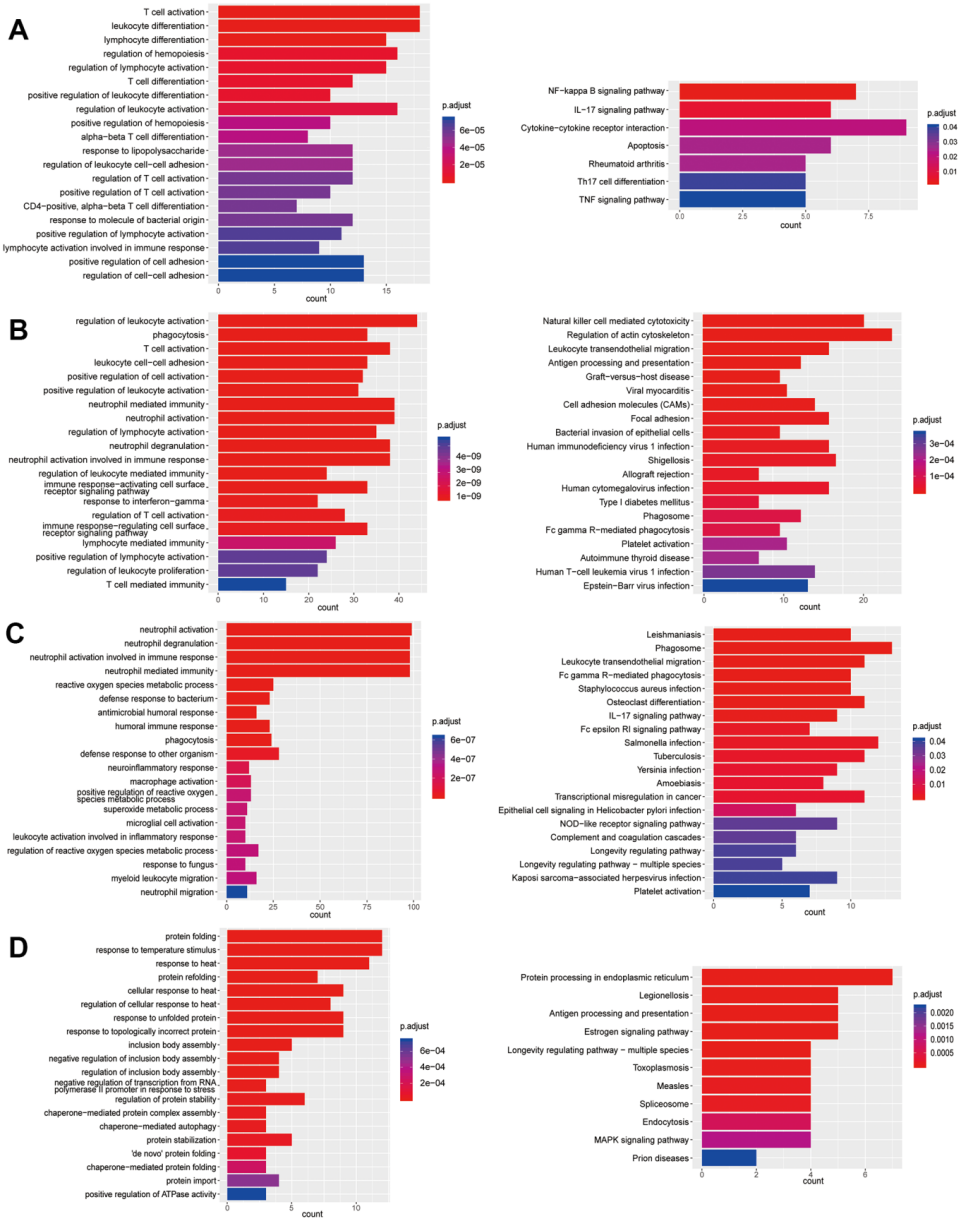
**Enrichment analysis of subtypes in T cells (T4-T7).** (**A**) GO (left) and KEGG (right) enrichment analysis of DEGs in T4. (**B**) GO (left) and KEGG (right) enrichment analysis of DEGs in T5. (**C**) GO (left) and KEGG (right) enrichment analysis of DEGs in T6. (**D**) GO (left) and KEGG (right) enrichment analysis of DEGs in T7.

**Figure 7:**
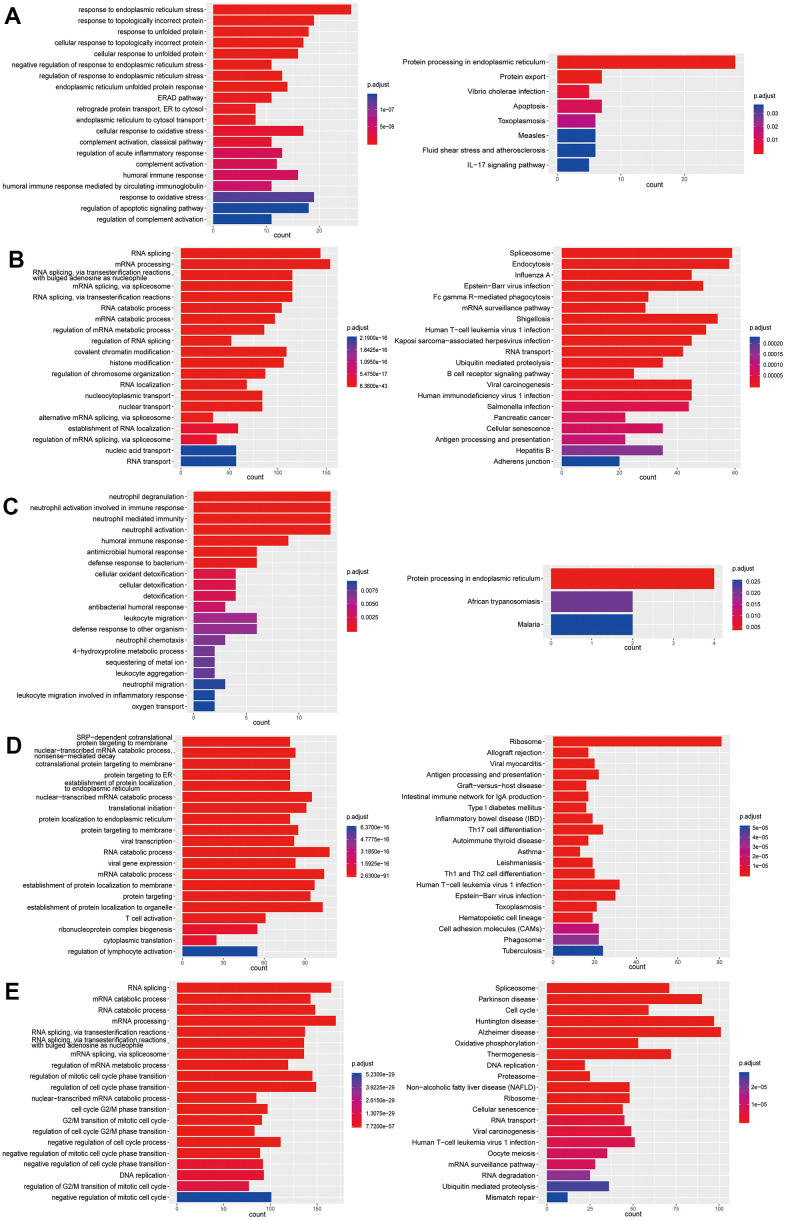
**Enrichment analysis of subtypes in B cells (B1-B5).** (**A**) GO (left) and KEGG (right) enrichment analysis of DEGs in B1. (**B**) GO (left) and KEGG (right) enrichment analysis of DEGs in B2. (**C**) GO (left) and KEGG (right) enrichment analysis of DEGs in B3. (**D**) GO (left) and KEGG (right) enrichment analysis of DEGs in B4. (**E**) GO (left) and KEGG (right) enrichment analysis of DEGs in B5.

GO and KEGG enrichment analyses suggested that several of these subtypes were involved in the regulation of bone metabolism (Table 1 and Figures 4–7 and Supplementary Tables 8–13), including *IL1B*^+^ monocytes (M1), CD4 T cells (T1), *GZMK*^+^*CCL4L2*^+^ T cells (T2), *GZMK*^+^*CCR6*^+^ T cells (T4), *GZMB*^+^*GNLY*^+^ T cells (T5), *GZMK*^+^*CXCL8*^+^ T cells (T6), *DNTT*^+^/*MME*^+^ pre-B cell (only B5), and *MSHA1*^+^ mature/activated B cell (B4).

**Table 1:** Enrichment analysis of subtypes in monocytes, T cells and B cells.

**Subtype**	**ID**	**Description**	**GeneRatio**	**p.adjust**	**Gene symbol**
M1	GO:0001503	ossification	19/399	0.011	VCAN, ATP2B1, PTGS2, FGR, CTNNB1, TGFB1, H3F3A, HIF1A, AREG, TCIRG1, CEBPB, DDX5, DDX21, TPM4, SNAI1, IL6R, JUNB, CLEC5A, IL6
	GO:0001649	osteoblast differentiation	14/399	0.006	VCAN, CTNNB1, H3F3A, AREG, TCIRG1, CEBPB, DDX5, DDX21, TPM4, SNAI1, IL6R, JUNB, CLEC5A, IL6
	GO:0030316	osteoclast differentiation	10/399	0.001	LILRB3, FCER1G, CTNNB1, MAFB, TGFB1, OSCAR, TCIRG1, CEBPB, ANXA2, JUNB
	GO:0045453	bone resorption	5/399	0.045	CTNNB1, ADAM8, TNFAIP3, TCIRG1, IL6
	GO:0046849	bone remodeling	6/399	0.048	CTNNB1, TGFB1, ADAM8, TNFAIP3, TCIRG1, IL6
	hsa04380	osteoclast differentiation	19/238	0.000	IL1B, NCF2, LILRB2, LILRA5, LILRB3, FOSL2, NFKBIA, SOCS3, TGFB1, IFNGR2, LCP2, OSCAR, NFKB2, NCF1, NFKB1, IFNGR1, IL1A, FYN, JUNB
T1	GO:0030316	osteoclast differentiation	4/62	0.007	GPR183, JUNB, FOS, FOXP1
T2	GO:0030316	osteoclast differentiation	6/191	0.008	PIK3R1, CD81, CCL3, IFNG, GNAS, TGFB1
	GO:0045670	regulation of osteoclast differentiation	4/191	0.046	PIK3R1, CCL3, IFNG, GNAS
T4	GO:0030316	osteoclast differentiation	4/109	0.037	IL23R, CA2, GPR183, FOS
	GO:0045672	positive regulation of osteoclast differentiation	3/109	0.010	IL23R, CA2, FOS
T5	GO:0030316	osteoclast differentiation	6/308	0.043	TYROBP, FCER1G, TGFB1, CCL3, CD81, CEBPB
	GO:0045778	positive regulation of ossification	6/308	0.043	TGFB1, IFITM1, CLIC1, ADRB2, ZBTB16, CEBPB
T6	GO:0030316	osteoclast differentiation	7/246	0.007	LTF, FCER1G, TYROBP, LILRB3, SNX10, MAPK14, FOS
B5	GO:0001649	osteoblast differentiation	32/1647	0.042	LEF1, CDK6, H3F3A, HNRNPC, SMAD1, CBFB, ID2, ID3, HNRNPU, ATP5F1B, RBMX, FBXO5, SYNCRIP, MEF2D, GNAS, SNRNP200, CLTC, ALYREF, REST, HDAC7, DHX9, DDX5, MEF2C, CLIC1, H3F3B, CTNNB1, ADAR, TPM4, RPS15, FBL, LIMD1, PHB
B4	hsa04380	osteoclast differentiation	13/383	0.040	JUNB, JUND, NFKB2, NFATC1, GRB2, TGFB1, FOS, FOSB, CYLD, PPP3CA, SOCS3, NCF1, NFKBIA

Monocytes: M1-M3; T cells: T1-T7; B cells: B1-B5.

### Complex inter-cellular communication networks in human femoral head

**Figure 8:**
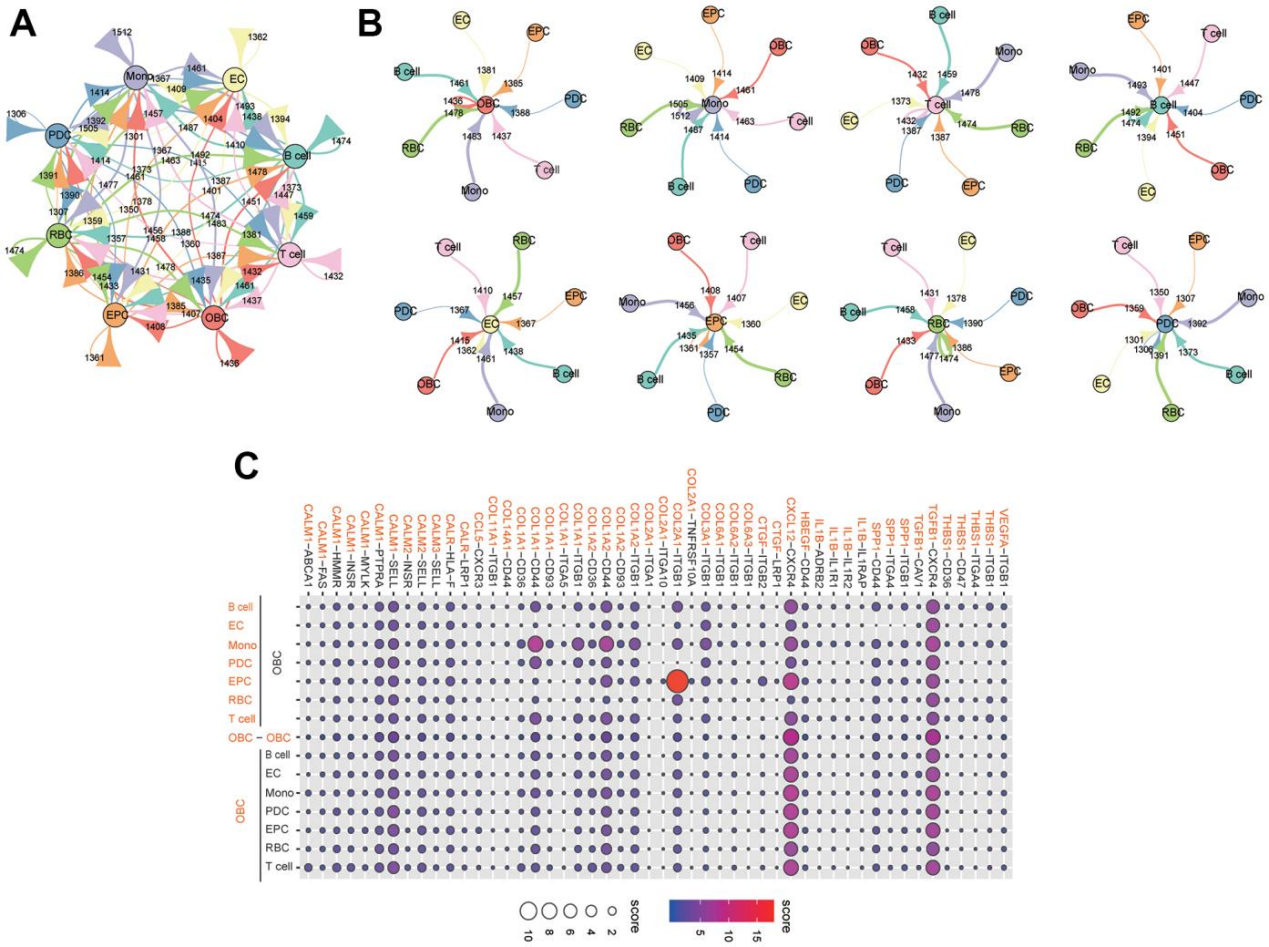
**Extensive crosstalk networks in human femoral head tissue cells.** (**A**) Capacity for inter-cellular communication between osteoblastic lineage cells and other cells in human femoral head. The map quantifies the potential communication, but does not consider the anatomical location or boundaries of the cell type. The color of each line indicates the ligands expressed by the same color cell type. The lines connect to the cell clusters types that express the cognate receptors. The thickness of line is proportional to the number of ligands. The loop indicates autocrine circuits. The number indicates the quantity of ligand-receptor pairs in each inter-cellular link. (**B**) Detailed view of the ligands and cognate receptors between each cell type. (**C**) Overview of selected ligand-receptor interactions of osteoblastic lineage cells. Interaction score is indicated by circle size and color. Mono: monocyte; EC: endothelial cell; OBC: osteoblastic lineage cell; EPC: endothelial progenitor cell; RBC: red blood cell; PDC: plasmacytoid dendritic cell.

We identified ligand-receptor pairs and molecular interactions among bone tissue cells (except granulocytes) (Figure 8A). Cognate receptors with broadcast ligands were detected, demonstrating extensive communication between osteoblastic lineage cells and other types of cells (Figure 8A, 8B). Our results suggested that chemokine, transforming growth factor and collagen had significant roles in inter-cellular communications (Figure 8C). The "CXCL12-CXCR4" interaction pair played important role in the crosstalks between bone tissue cells. Previous studies have reported that the CXCL12/CXCR4 signaling was involved in the regulation of bone homeostasis [19–21]. Notably, the "COL2A1-ITGB1" interaction pair was significant in the crosstalk between EPCs and osteoblastic lineage cells (Figure 8C). Additionally, compared with other cells, monocytes could act through COL1A1-/COL1A2-CD44 interaction pairs to perform closer cell communication (measured with interaction score) with osteoblastic lineage cells (Figure 8C).

## DISCUSSION

Bone is a complex tissue and undergoes modeling/remodeling constantly during life [22]. Various types of cells are involved in the regulation of bone homeostasis, such as bone cells, stromal cell, immune cells, endothelial cells, which also communicate with each other closely [4]. Therefore, it is fundamental to study the cellular composition of the bone tissue cells and the crosstalks between them. In this study, we applied scRNA-seq analyses on freshly isolated bone tissue cells from human femoral head. We identified two novel bone metabolism-related genes, *PRSS23* and *MXRA8*. We discovered several subtypes of immune cells (monocytes, T cells and B cells) that may be involved in the regulation of bone metabolism. Finally, the cell-cell communication analysis suggested complex inter-cellular communication networks among human femoral head tissue cells, and the close crosstalk between EPCs and osteoblastic lineage cells via the "COL2A1-ITGB1" interaction pair. Our results provided an initial systematic dissection of human femoral head tissue at single-cell resolution and a global single-cell profile of how different cells work together in human femoral head.

To avoid potential alternation of transcriptome profiles caused by *in vitro* operations (e.g. culturing) [23], we performed scRNA-seq on the freshly isolated primary femoral head tissue cells. In addition, we identified two novel bone metabolism-related genes, *PRSS23* and *MXRA8*, by analyzing the PPI network constructed from DEGs in osteoblastic lineage cells, and showed that the expression of these two genes were significantly increased during *in vitro* osteogenic differentiation. Based on our recent scRNA-seq data of BM-MSCs [24] and osteoblasts [25], *PRSS23* was highly expressed in the osteoblast precursors and early osteoblasts (pre-osteoblast and undetermined osteoblast). This result suggested that *PRSS23* may promote the differentiation of BM-MSCs into osteoblasts. Previous studies reported that, in breast cancer cells, *PRSS23* was co-expressed with estrogen receptor α (ERα), and *PRSS23* knockdown may suppress estrogen-driven cell proliferation of breast cancer cells [26]. Since estrogens were highly significant for bone metabolism and maintaining bone mineral density (BMD) [27], we speculated that *PRSS23* may regulate bone metabolism through affecting ERα gene expression. Additionally, *PRSS23* have been reported to interact with TGFB signaling pathways [28], and TGFB signaling pathway was significant for bone metabolism [29]. Therefore, we speculated that *PRSS23* may also regulate bone metabolism through mediating the TGFB signaling pathway. *MXRA8* was highly expressed in both BM-MSCs and osteoblasts (Figure 2D), suggesting that it may play a critical role in maintaining the activity and function of BM-MSCs and osteoblasts. Recent studies showed that *MXRA8* was a lipid metabolism-related gene [30] and also related to the proliferation of growth plate chondrocytes [31]. Interestingly, MXRA8 is a cell adhesion molecule, as an entry mediator for arthritogenic alphaviruses [32], and arthritogenic alphaviruses would cause chronic musculoskeletal disease [33]. Taken together, *PRSS23* and *MXRA8* were likely related to bone metabolism in humans.

In the monocytes, we found the *IL1B*^+^ monocytes (M1) could regulate bone metabolism, and this subtype of monocytes have been reported as a key potential mediator of the pathogenesis of rheumatoid arthritis [34]. Besides, the *IL1B*^+^ monocytes (M1) contain the *TRAP*^+^*CTSK*^+^ osteoclasts precursor (Figure 3J). Among the T cells, we found the majority of CD8 T cells in bone tissue express *GZMK*, which is similar to the results in synovial tissue [34]. GO enrichment analysis suggested that *GZMK*^+^*CCR6*^+^CD8 T cells (T4) could promote osteoclastogenesis and enhance bone resorption. *CCL20* was highly expressed in *GZMK*^+^*CCR6*^+^CD8 T cells (T4) (Figure 3K), and *CCL20* can enhance osteoclastogenesis and induce osteoclast differentiation [35, 36]. In contrast, another subpopulation of CD8 T cells, *GZMK*^+^*CXCL8*^+^ T cells (T6), specifically express high levels of *LTF* (Figure 3K), which can inhibit the bone resorption mediated by osteoclasts [37]. Therefore, the previous notion that CD8 T cells are suppressive to bone resorption should be re-evaluated at single-cell level [38–40]. In addition, GO enrichment analysis suggested that the pre-B cells (only B5) may also regulate the differentiation of osteoblasts. Therefore, future studies are needed to further explore the functional roles of *GZMK*^+^*CCR6*^+^CD8 T cells (T4) and pre-B cells (only B5) on bone metabolism in the context of their *in vivo* functional importance in bone tissues.

To explore inter-cellular interaction in human femoral head tissue cells, we constructed the inter-cellular communication networks in femoral head based on known ligand-receptor interactions. In the network, EPCs closely communicated with osteoblastic lineage cells via "COL2A1-ITGB1" interaction. Since COL2A1 is a known chondrogenic marker [41], we suspected that EPCs may regulate chondrogenesis of osteoblastic lineage cells through "COL2A1-ITGB1" interaction. For novel bone metabolism-related genes, *PRSS23* and *MXRA8*, we did not find any significant crosstalks in the cell-cell communication analysis, which is probably due to the very limited knowledge about the functions of these two genes.

Despite interesting and novel findings in this initial comprehensive characterization of cells and their interactions *in vivo* in human femoral head at single cell level, our study may have some limitations. First, due to limited amount of data (only 691 osteoblastic lineage cells detected in our dataset), we were unable to further dissect subpopulations of osteoblastic lineage cells. This is mainly because we performed scRNA-seq on the primary femoral head tissue cells without any purification/enrichment procedures specifically for osteoblastic lineage cells. Also, the samples of this study were from subjects with osteoporosis or osteopenia because appropriate bone samples can be obtained from these subjects during hip replacement therapy, and this might incur some bias in the cell subpopulation identification and proportion estimation compared with healthy individuals. Previous studies showed that the number and activity of osteoblastic lineage cells were significantly decreased in people with osteoporosis or osteopenia [42]. Therefore, we need to specifically isolate/enrich osteoblastic lineage cells from study subject with different health conditions in future studies. As the osteoblastic lineage cells are a heterogeneous cells population, a negative sorting approach can be adopted [43]. In addition, we were unable to detect osteoclasts in this study. This is as expected because the size of osteoclasts (150-200 μm in diameter) exceeds the upper limit of cell size (40 μm in diameter) compatible with the current 10x Genomics system, and thus osteoclasts were filtered out before the scRNA-seq library construction. In addition, osteoclasts are multinucleated cells with heterogeneous nuclei inside mature osteoclasts, and thus the current single-nucleus RNA-seq is not suitable for osteoclasts either [44]. In future studies, spatial transcriptomics may hopefully provide an opportunity to explore the cellular heterogeneity of osteoclasts and the relationship with bone metabolism [45].

In summary, our study characterized the cellular composition of the human femoral head tissue cells, and identified *PRSS23* and *MXRA8* as novel bone metabolism-related genes. The complex inter-cellular communication networks in human femoral head suggest that various types of cells are involved in the regulation of bone metabolism, and EPCs communicate with osteoblastic lineage cells closely via the "COL2A1-ITGB1" interaction pair. Our study provides a systematic dissection of human femoral head at the single-cell level, and shows the global single-cell profile of how different cells work together in human femoral head on the single-cell resolution, which is a unique resource for in-depth insights into bone metabolism.

In future studies, more subjects should be included to further dissect subpopulation of osteoblastic lineage cells, and to explore how different health states affect the bone metabolism and vice versa. Besides, by combining scRNA-seq with spatial transcriptomics [45] and scATAC-seq (a powerful tool to evaluate chromatin accessibility at the single-cell level) [46], we will aim to unveil the complicated crosstalk between bone tissue cells, and the gene regulatory network within/between them. In the meantime, deconvolution of the cellular heterogeneity of bone tissue cells *in vivo* in humans represents an important and necessary advancement step towards improving our understanding of bone physiological processes.

## MATERIALS AND METHODS

### Study subjects

The study was approved by the Medical Ethics Committee of Xiangya Hospital of Central South University and written informed consent was obtained from all participants. The study subjects consisted of four Chinese subjects of Han ethnicity (detailed information of the study subjects provided in Supplementary Table 1), who underwent hip replacement surgery at Xiangya Hospital of Central South University. All the subjects were screened with a detailed questionnaire, medical history, physical examination, and measured for bone mineral density (BMD) before surgery. Subjects were excluded from the study if they had chronic diseases that may affect bone metabolism, including but not limited to renal failure, liver failure, diabetes mellitus, hematologic diseases, malabsorption syndrome, disorders of the thyroid/parathyroid, malignant tumors, ankylosing spondylitis, hyperprolactinemia, oophorectomy, or previous pathological fractures [47]. The femur head was collected from the patient during hip replacement surgery. The specimens were immediately stored at 4° C temporarily and transferred to the wet laboratory within 2 hours, where they were processed within 24 hours after delivery.

### BMD measurement

BMD (g/cm^2^) was measured by the duel energy x-ray absorptiometry (DXA) fan-beam bone densitometer (Hologic QDR 4500A, Hologic, Inc., Bedford, MA, USA) at the right hip (femoral neck and trochanter) and the lumbar spine (L1-L4). According to the World Health Organization (WHO) definition [48] and the BMD reference established for Chinese [49], subject with T-score ≤ -2.5 is clinically diagnosed as osteoporosis, while T-score between -2.5 and -1 as osteopenia, and T-score > -1.0 are considered healthy.

### Isolation of bone tissue cells

Bone tissue cells were extracted from the femoral head specimens based on widely used dissociation protocols with a few adjustments [50, 51]. First, femoral heads were washed three times with αMEM (Cat: SH30265.01, HyClone, USA) and dissected into small pieces of approximately 1-2 mm in diameter. Bone pieces (10 g wet weight) were placed into a 50 ml conical tube with 20 ml of 2 mg/ml collagenase type II (Cat: A004174-0001, Sangon Biotech, China) dissolved in αMEM with 100 U/ml Penicillin and 100μg/ml Streptomycin (Cat: 15140-122, Gibco, USA) and digested with gentle agitation for 25 minutes at 37° C. After that, the collagenase solution was aseptically removed and bone pieces were rinsed in 10 ml PBS for 3 times. Briefly, after five rounds of digestion, we combined the collagenase solutions from the last two rounds of digestion and filtered the solution through a 40 μm filter. Finally, we incubated the collected cells with red blood cell (RBC) lysis buffer (Cat: R1010, Solarbio, China) for 5 minutes and then washed it twice with PBS.

### scRNA-seq library preparation and sequencing

scRNA-seq libraries were prepared using Single Cell 3’ Library Gel Bead Kit V3 following the manufacturer’s guidelines (https://support.10xgenomics.com/single-cell-gene-expression/library-prep/doc/user-guide-chromium-single-cell-3-reagent-kits-user-guide-v3-chemistry). Single cell 3’ Libraries contain the P5 and P7 primers used in Illumina bridge amplification PCR. The 10x Barcode and Read 1 (primer site for sequencing read 1) were added to the molecules during the GEM-RT incubation. The P5 primer, Read 2 (primer site for sequencing read 2), Sample Index and P7 primer were added during library construction. The protocol was designed to support library construction from a wide range of cDNA amplification yields spanning from 2 ng to > 2 μg without modification. Finally, scRNA-seq libraries were sequenced on the Illumina Novaseq6000 platform with a sequencing depth of at least 100,000 reads per cell for a 150bp paired end (PE150) run.

### Pre-processing of scRNA-seq data

The FASTQ files were mapped to the human transcriptome (GRCh38/hg38) using Cell Ranger 3.0 (https://support.10xgenomics.com/single-cell-gene-expression/software/pipelines/latest/what-is-cell-ranger). To create Cell Ranger-compatible reference genomes, the references were rebuilt according to instructions from 10x Genomics (https://www.10xgenomics.com), which performs alignment, filtering, barcode counting and UMI counting. Finally, the digital gene expression matrix was generated. For quality control (QC), we used the R (version 3.6.1, https://www.r-project.org/) and *Seurat* R package (version 3.1, https://satijalab.org/seurat/) [52, 53] to calculate the distribution of genes detected per cell and remove the cells in the top or the bottom 2% quantile. We also excluded cells in which more than 10% of the transcripts were attributed to mitochondrial genes.

### Dimension reduction and cluster identification

To visualize and cluster the data, we selected top 2,000 most variable genes for principal-component analysis (PCA), and then, we used the first 20 principal-components (PCs) for visualization by t-Distributed Stochastic Neighbor Embedding (t-SNE) [54]. Next, we performed an unbiased graph-based method for clustering analysis using the first 20 PCs [55]. To identify differentially expressed genes (DEGs) between clusters, Wilcoxon rank-sum test was used to identify genes showing significantly higher levels of expression (false discovery rate (FDR) < 0.05) in a specific cluster compared to the other clusters.

### Pathway enrichment analysis

To investigate the biological processes and signal pathways associated with cell type, we performed gene ontology (GO) and Kyoto Encyclopedia of Genes and Genomes (KEGG) enrichment analyses for the genes that were identified as important DEGs for clusters (adjusted *p* value < 0.05), by using the *clusterProfiler* R package [56].

### Protein-protein interaction (PPI) network, hub genes and module analysis

To identify the most significant gene among the DEGs in the context of functioning in gene networks, a PPI network of DEGs (selected with average log(Fold change) > 1.0, adjusted *p* value < 0.05) was constructed using the Search Tool for the Retrieval of Interacting Genes (STRING, http://string.embl.de/) [57]. The Cytoscape software (version 3.7.2) was applied to visualize and analyze the molecular interaction networks [58]. The hub genes of the PPI network were identified by cytoHubba (Cytoscape) [59]. The modules of the PPI network were selected by Molecular Complex Detection (MCODE) (Cytoscape) [60]. The biological analyses of hub genes were constructed using BiNGO (Cytoscape) [61].

### Cell-cell communication analysis

To explore the potential interactions between cells in human femoral head, we used *iTALK* [62] to perform cell-cell communication analysis, which is an R toolkit for visualizing ligand-receptor-mediated inter-cellular interaction in scRNA-seq data. The product of average receptor expression and average ligand expression was calculated in each cell cluster to score the enriched receptor-ligand interactions.

### Public datasets

We recently generated scRNA-seq datasets of human BM-MSCs and human osteoblasts [24, 25], which can be accessed from GEO database (https://www.ncbi.nlm.nih.gov/geo/) [63] under the accession numbers of GSE147287 and GSE147390, respectively. In this study, we processed these datasets using the same parameters as described in our previous studies [24, 25]. The gene expression profile of osteogenic differentiation by BM-MSCs *in vitro* was obtained from the GEO database with accession numbers GSE37558 [64]. And the data were log_2_ transformed and normalized using the quantile-normalization approach.

**Figure 9:**
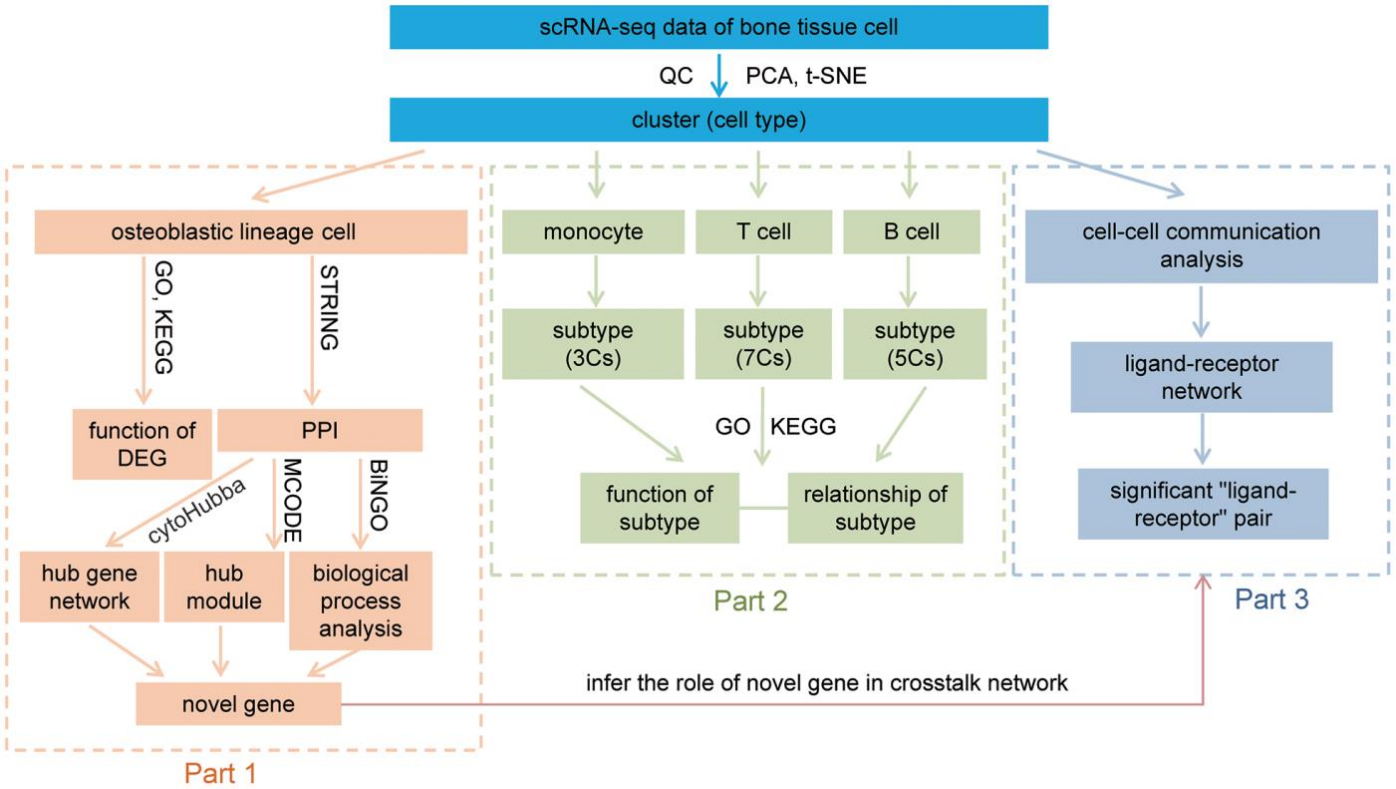
**Workflow of this study.** After QC, dimension reduction, and clustering of the data, we identify nine cell types in our data. The downstream analysis was divided into three parts. Part 1, analysis of osteoblastic lineage cells, functional analyses of osteoblastic lineage cells and identify novel bone metabolism-related gene. Part 2, revealing distinct subtypes in monocytes, T cells and B cells, and discussion their relationship with bone metabolism. Part 3, constructing the communication networks of human femoral head tissue cells, and inferring the role of novel metabolism-related gene in crosstalk network. QC: quality control; PCA: principal-component analysis; t-SNE: t-Distributed Stochastic Neighbor Embedding; GO: gene ontology enrichment analysis; KEGG: Kyoto Encyclopedia of Genes and Genomes enrichment analysis; DEG: differentially expressed gene; PPI: protein-protein interaction; MCODE: Molecular Complex Detection; Cs: clusters.

The data analyses of this study were divided into three parts, and illustrated in Figure 9.

### Ethics approval

The study was approved by the Medical Ethics Committee of Xiangya Hospital of Central South University, and the IRB approval number is No. 201912315.

### Consent to participate

Written informed consent was obtained from all participants.

### Consent for publication

All authors gave their consent for publication.

### Availability of data and material

The scRNA-seq data of human femoral head tissue cells from four human samples is available in the GEO database with accession numbers GSE169396. The scRNA-seq data of human BM-MSCs and human osteoblasts are available in the GEO database with accession numbers GSE147287 and GSE108891. The data of osteogenic differentiation by BM-MSCs *in vitro* was obtained from the GEO database with accession numbers GSE37558.

## Supplementary Material

Supplementary Figures

Supplementary Table 1

Supplementary Table 2

Supplementary Table 3

Supplementary Table 4

Supplementary Table 5

Supplementary Table 6

Supplementary Table 7

Supplementary Table 8

Supplementary Table 9

Supplementary Table 10

Supplementary Table 11

Supplementary Table 12

Supplementary Table 13
