# Supplementary material for: Single-cell RNA sequencing of human femoral head in vivo
Source: Aging (Albany NY). 2021 Jun 10;13(11):15595–619. doi: 10.18632/aging.203124 (PMC8221309; doi:10.18632/aging.203124)
Supplement: Supplementary Figures [file aging-13-203124-s001.pdf]

## SUPPLEMENTARY FIGURES

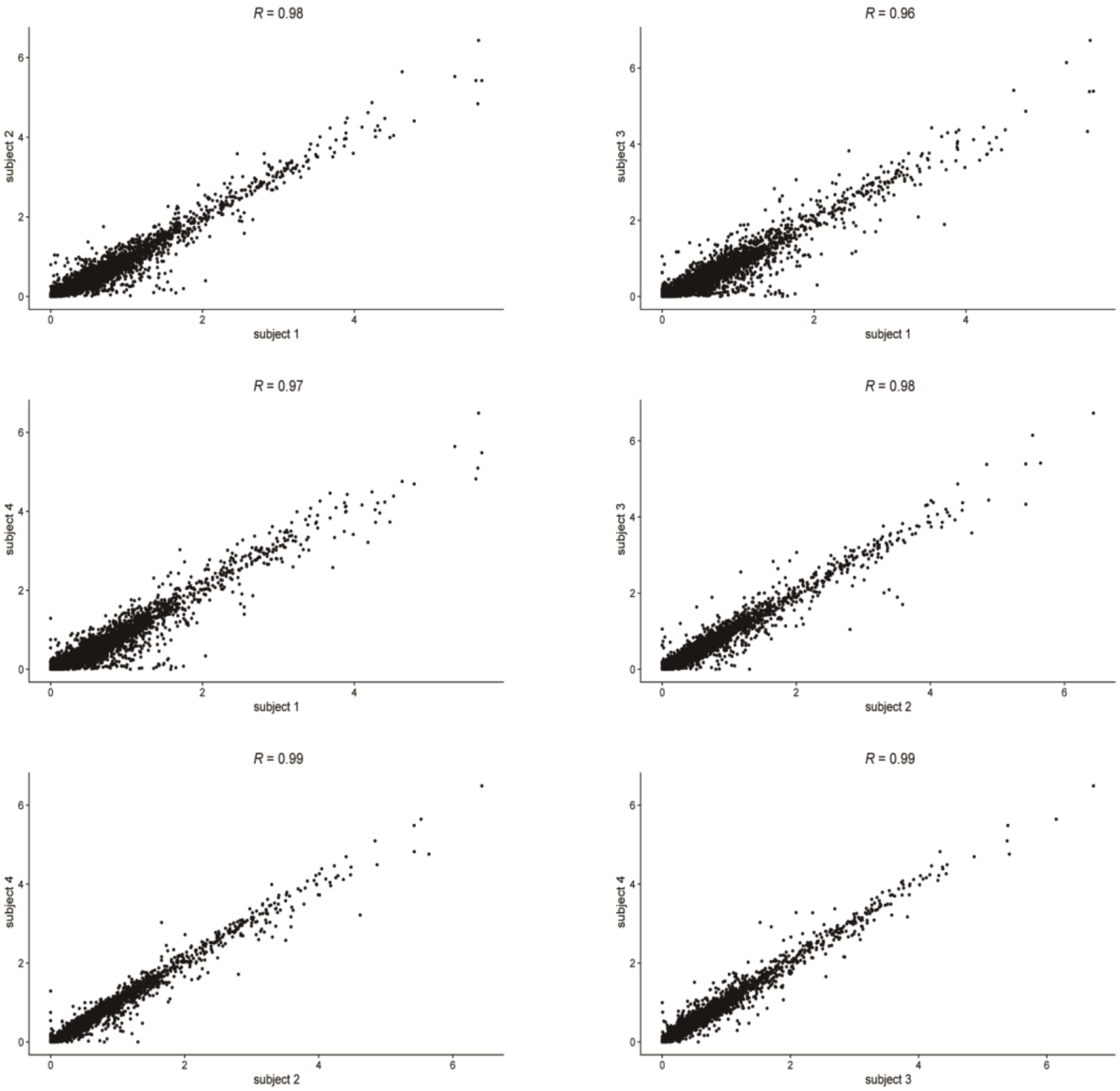


**Supplementary Figure 1. Correlation of gene expression profiles between each two subjects.** Each dot represents an individual gene.

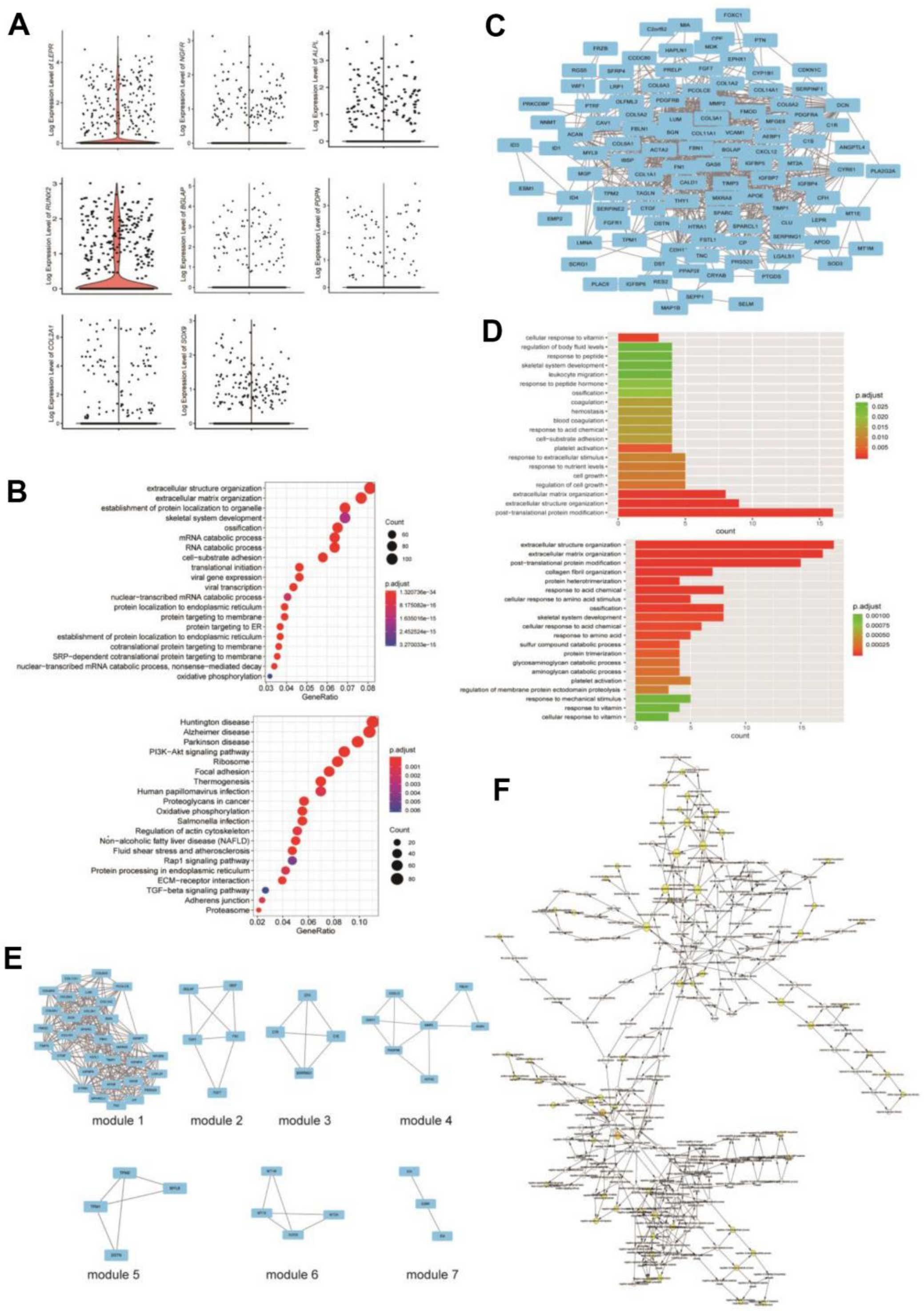


**Supplementary Figure 2. PPI network and module analysis for DEGs of osteoblastic lineage cells.** (**A**) Violin plots show the expression of marker genes about BM-MSCs (*LEPR*, *NGFR*), osteoblasts (*ALPL*, *RUNX2*, *BGLAP*), osteocytes (*PDPN*), and chondrocytes (*COL2A1*, *SOX9*) in the osteoblastic lineage cells. Each dot represents one cell. (**B**) GO (above) and KEGG (below) enrichment analysis for DEGs of osteoblastic lineage cells. (**C**) Visualize PPI network with Cytoscape. The PPI network consists of 111 nodes and 800 edges. (**D**) GO enrichment analysis of the 20 hub genes with a higher degree of connectivity in gene network (above). GO enrichment analysis of genes in module 1 (below). (**E**) The geometry view of seven modules. (**F**) The biological process analysis of hub genes in module 1 was constructed using BiNGO (Cytoscape). The color depth of node refers to the corrected *p* value of ontologies. The size of node refers to the number of genes that are involved in the ontologies. The corrected *p* value < 0.05 was considered statistically significant.
