# Supplementary material for: Single-cell RNA sequencing of human femoral head in vivo
Source: Aging (Albany NY). 2021 Jun 10;13(11):15595–619. doi: 10.18632/aging.203124 (PMC8221309; doi:10.18632/aging.203124)
Supplement: Supplementary Table 1 [file aging-13-203124-s002.pdf]

Supplementary Table 1. The detailed information of this study subjects.

ID	Age	Gender	Sample	T-score of BMD		Disease state	Date
				Lumbar vertebra	Left hip joint		
S1	61	female	femoral head	-3	-1.9	osteoarthritis and osteoporosis	2019.7.16
S2	45	female	femoral head	-1.3	-1.2	osteoarthritis and osteopenia	2019.7.19
S3	66	male	femoral head	NA	NA	NA	2019.7.26
S4	31	male	femoral head	0.6	-1.1	osteoarthritis and osteopenia	2019.8.7

S3 without bone mineral density test due to the tight schedule of surgery.
