# Supplementary material for: Single-cell RNA sequencing of human femoral head in vivo
Source: Aging (Albany NY). 2021 Jun 10;13(11):15595–619. doi: 10.18632/aging.203124 (PMC8221309; doi:10.18632/aging.203124)
Supplement: Supplementary Table 5 [file aging-13-203124-s006.pdf]

Supplementary Table 5. The detailed information of each module.

Module	Score (Density*#Nodes)	Nodes	Edges	Node IDs
1	19.097	32	296	CTGF, PRSS23, TNC, BGN, TIMP3, FSTL1, FBN1, APOE, LGALS1, SPARCL1, IGFBP4, COL6A3, SPARC, MXRA8, DCN, LUM, MFGE8, COL3A1, GAS6, COL5A2, TIMP1, COL6A2, COL6A1, PCOLCE, IGFBP7, COL1A1, CYR61, COL1A2, FMOD, CP, COL11A1, IGFBP5
2	4	5	8	FN1, FGF7, IBSP, BGLAP, THY1
3	4	4	6	SERPING1, C1S, C1R, CFH
4	3.667	7	11	CDH11, MMP2, PDGFRB, ACAN, FBLN1, CXCL12, ACTA2
5	3.333	4	5	TPM1, MYL9, TPM2, DSTN
6	3.333	4	5	MT1E, SOD3, MT2A, MT1M
7	3	3	3	ID4, ID3, ESM1
