# Supplementary material for: Single-cell RNA sequencing of human femoral head in vivo
Source: Aging (Albany NY). 2021 Jun 10;13(11):15595–619. doi: 10.18632/aging.203124 (PMC8221309; doi:10.18632/aging.203124)
Supplement: Supplementary Table 7 [file aging-13-203124-s008.pdf]

Supplementary Table 7. Function of the top 16 hub genes of module 1 in bone metabolism.

Gene symbol	Full name	Function
PRSS23	*serine protease 23*	NA
TNC	*tenascin C*	An extracellular matrix glycoprotein involved in osteogenesis and bone mineralization [1] .
FSTL1	*follistatin like 1*	Promotes chondrocyte apoptosis [2] and osteoclast formation [3].
FBN1	*fibrillin 1*	Limits osteoclast formation and function [4]; a negative regulators of bone resorption [5].
APOE	*apolipoprotein E*	Plays crucial roles in maintaining bone mass by promoting osteoblast differentiation and suppressing osteoclast differentiation [6].
LGALS1	*galectin 1*	Relates to osteoblast maturation [7], and plays a role in cell-cell and cell-matrix interactions of osteoblastic cells [8].
SPARCL1	*SPARC like 1*	An extracellular matrix remodel gene [9]; a member of the osteonectin family of proteins [10]; suppresses osteosarcoma metastasis [11].
IGFBP4	*insulin like growth factor binding protein 4*	Highly expressed in adipocytes and osteoblasts [12]; regulates bone metabolism [13–15].
MXRA8	*matrix remodeling associated protein 8*	NA
MFGE8	*milk fat globule EGF and factor V/VIII domain containing*	Regulates osteoclast homeostasis and inflammatory bone loss [16].
GAS6	*growth arrest specific 6*	Enhances the bone resorbing activity of mature osteoclasts [17]; induces osteoclast differentiation [18].
TIMP1	*TIMP metallopeptidase inhibitor 1*	Inhibits the activity of MMPs and then regulate the degradation of bone extracellular matrix molecules [19].
IGFBP7	*insulin like growth factor binding protein 7*	Inhibits osteoclastogenesis and osteoclast activity [20]; enhanced osteogenic differentiation of BM-MSCs *in vitro* and promoted new bone formation *in vivo* [21].
CYR61	*cysteine-rich protein 61*	Modulates mature osteoblast and osteocyte function to regulate bone mass [22]; stimulates proliferation and differentiation of osteoblasts *in vitro* and contribute to bone remodeling *in vivo* in myeloma bone disease [23]; regulates adipocyte differentiation from mesenchymal stem cells [24].
CP	*ceruloplasmin*	Inhibits osteoblast activity, mineralization [25, 26].
IGFBP5	*insulin like growth factor binding protein 5*	The IGFBP5 produced by osteoblasts stimulates osteoclastogenesis and bone resorption, and as an osteoblast-osteoclast coupling factor [27].

SPARC: secreted protein acidic and cysteine rich. TIMP: tissue inhibitor of metalloproteases; MMPs: matrix metalloproteinases; BM-MSCs: bone marrow-derived mesenchymal stem cells.

REFERENCES

1. Li C, Cui Y, Luan J, Zhou X, Li H, Wang H, Shi L, Han J. Tenascin C affects mineralization of SaOS2 osteoblast-like cells through matrix vesicles. Drug Discov Ther. 2016; 10:82–87. https://doi.org/10.5582/ddt.2016.01009 PMID:26961327

2. Xu C, Jiang T, Ni S, Chen C, Li C, Zhuang C, Zhao G, Jiang S, Wang L, Zhu R, van Wijnen AJ, Wang Y. FSTL1 promotes nitric oxide-induced chondrocyte apoptosis via activating the SAPK/JNK/caspase3 signaling pathway. Gene. 2020; 732:144339. https://doi.org/10.1016/j.gene.2020.144339 PMID:31927008

3. Kim HJ, Kang WY, Seong SJ, Kim SY, Lim MS, Yoon YR. Follistatin-like 1 promotes osteoclast formation via RANKL-mediated NF-κB activation and M-CSF-induced precursor proliferation. Cell Signal. 2016; 28:1137–44. https://doi.org/10.1016/j.cellsig.2016.05.018 PMID:27234130

4. Tiedemann K, Boraschi-Diaz I, Rajakumar I, Kaur J, Roughley P, Reinhardt DP, Komarova SV. Fibrillin-1 directly regulates osteoclast formation and function by a dual mechanism. J Cell Sci. 2013; 126:4187–94. https://doi.org/10.1242/jcs.127571 PMID:24039232

5. Nistala H, Lee-Arteaga S, Smaldone S, Siciliano G, Ramirez F. Extracellular microfibrils control osteoblast-supported osteoclastogenesis by restricting TGF{beta} stimulation of RANKL production. J Biol Chem. 2010; 285:34126–33.

https://doi.org/10.1074/jbc.M110.125328
PMID:20729550

6. Noguchi T, Ebina K, Hirao M, Otsuru S, Guess AJ, Kawase R, Ohama T, Yamashita S, Etani Y, Okamura G, Yoshikawa H. Apolipoprotein E plays crucial roles in maintaining bone mass by promoting osteoblast differentiation via ERK1/2 pathway and by suppressing osteoclast differentiation via c-Fos, NFATc1, and NF-κB pathway. Biochem Biophys Res Commun. 2018; 503:644–50.
https://doi.org/10.1016/j.bbrc.2018.06.055
PMID:29906458

7. Hopwood B, Tsykin A, Findlay DM, Fazzalari NL. Gene expression profile of the bone microenvironment in human fragility fracture bone. Bone. 2009; 44:87–101.
https://doi.org/10.1016/j.bone.2008.08.120
PMID:18840552

8. Tübel J, Saldamli B, Wiest I, Jeschke U, Burgkart R. Expression of the tumor markers sialyl Lewis A, sialyl Lewis X, Lewis Y, Thomsen-Friedenreich antigen, galectin-1 and galectin-3 in human osteoblasts *in vitro*. Anticancer Res. 2012; 32:2159–64.
PMID:22593503

9. Mintz MB, Sowers R, Brown KM, Hilmer SC, Mazza B, Huvos AG, Meyers PA, Lafleur B, McDonough WS, Henry MM, Ramsey KE, Antonescu CR, Chen W, et al. An expression signature classifies chemotherapy-resistant pediatric osteosarcoma. Cancer Res. 2005; 65:1748–54.
https://doi.org/10.1158/0008-5472.CAN-04-2463
PMID:15753370

10. Hashimoto N, Sato T, Yajima T, Fujita M, Sato A, Shimizu Y, Shimada Y, Shoji N, Sasano T, Ichikawa H. SPARCL1-containing neurons in the human brainstem and sensory ganglion. Somatosens Mot Res. 2016; 33:112–17.
https://doi.org/10.1080/08990220.2016.1197115
PMID:27357901

11. Zhao SJ, Jiang YQ, Xu NW, Li Q, Zhang Q, Wang SY, Li J, Wang YH, Zhang YL, Jiang SH, Wang YJ, Huang YJ, Zhang XX, et al. SPARCL1 suppresses osteosarcoma metastasis and recruits macrophages by activation of canonical WNT/β-catenin signaling through stabilization of the WNT-receptor complex. Oncogene. 2018; 37:1049–61.
https://doi.org/10.1038/onc.2017.403
PMID:29084211

12. Boney CM, Moats-Staats BM, Stiles AD, D'Ercole AJ. Expression of insulin-like growth factor-I (IGF-I) and IGF-binding proteins during adipogenesis. Endocrinology. 1994; 135:1863–68.
https://doi.org/10.1210/endo.135.5.7525256
PMID:7525256

13. Miyakoshi N, Qin X, Kasukawa Y, Richman C, Srivastava AK, Baylink DJ, Mohan S. Systemic administration of insulin-like growth factor (IGF)-binding protein-4 (IGFBP-4) increases bone formation parameters in mice by increasing IGF bioavailability via an IGFBP-4 protease-dependent mechanism. Endocrinology. 2001; 142:2641–48.
https://doi.org/10.1210/endo.142.6.8192
PMID:11356715

14. Zhang M, Faugere MC, Malluche H, Rosen CJ, Chernausek SD, Clemens TL. Paracrine overexpression of IGFBP-4 in osteoblasts of transgenic mice decreases bone turnover and causes global growth retardation. J Bone Miner Res. 2003; 18:836–43.
https://doi.org/10.1359/jbmr.2003.18.5.836
PMID:12733722

15. Maridas DE, DeMambro VE, Le PT, Nagano K, Baron R, Mohan S, Rosen CJ. IGFBP-4 regulates adult skeletal growth in a sex-specific manner. J Endocrinol. 2017; 233:131–44.
https://doi.org/10.1530/JOE-16-0673 PMID:28184001

16. Abe T, Shin J, Hosur K, Udey MC, Chavakis T, Hajishengallis G. Regulation of osteoclast homeostasis and inflammatory bone loss by MFG-E8. J Immunol. 2014; 193:1383–91.
https://doi.org/10.4049/jimmunol.1400970
PMID:24958900

17. Nakamura YS, Hakeda Y, Takakura N, Kameda T, Hamaguchi I, Miyamoto T, Kakudo S, Nakano T, Kumegawa M, Suda T. Tyro 3 receptor tyrosine kinase and its ligand, Gas6, stimulate the function of osteoclasts. Stem Cells. 1998; 16:229–38.
https://doi.org/10.1002/stem.160229 PMID:9617898

18. Ruiz-Heiland G, Zhao Y, Derer A, Braun T, Engelke K, Neumann E, Mueller-Ladner U, Liu Y, Zwerina J, Schett G. Deletion of the receptor tyrosine kinase Tyro3 inhibits synovial hyperplasia and bone damage in arthritis. Ann Rheum Dis. 2014; 73:771–79.
https://doi.org/10.1136/annrheumdis-2012-202907
PMID:23632195

19. Hatori K, Sasano Y, Takahashi I, Kamakura S, Kagayama M, Sasaki K. Osteoblasts and osteocytes express MMP2 and -8 and TIMP1, -2, and -3 along with extracellular matrix molecules during appositional bone formation. Anat Rec A Discov Mol Cell Evol Biol. 2004; 277:262–71.
https://doi.org/10.1002/ar.a.20007
PMID:15052653

20. Ye C, Hou W, Chen M, Lu J, Chen E, Tang L, Hang K, Ding Q, Li Y, Zhang W, He R. IGFBP7 acts as a negative regulator of RANKL-induced osteoclastogenesis and oestrogen deficiency-induced bone loss. Cell Prolif. 2020; 53:e12752.

https://doi.org/10.1111/cpr.12752
PMID:31889368

21. Zhang W, Chen E, Chen M, Ye C, Qi Y, Ding Q, Li H, Xue D, Gao X, Pan Z. IGFBP7 regulates the osteogenic differentiation of bone marrow-derived mesenchymal stem cells via Wnt/β-catenin signaling pathway. FASEB J. 2018; 32:2280–91.
https://doi.org/10.1096/fj.201700998RR
PMID:29242275

22. Zhao G, Huang BL, Rigueur D, Wang W, Bhoot C, Charles KR, Baek J, Mohan S, Jiang J, Lyons KM. CYR61/CCN1 Regulates Sclerostin Levels and Bone Maintenance. J Bone Miner Res. 2018; 33:1076–89.
https://doi.org/10.1002/jbmr.3394 PMID:29351359

23. Liu H, Peng F, Liu Z, Jiang F, Li L, Gao S, Wang G, Song J, Ruan E, Shao Z, Fu R. CYR61/CCN1 stimulates proliferation and differentiation of osteoblasts *in vitro* and contributes to bone remodeling *in vivo* in myeloma bone disease. Int J Oncol. 2017; 50:631–39.
https://doi.org/10.3892/ijo.2016.3815
PMID:28035364

24. Yang Y, Qi Q, Wang Y, Shi Y, Yang W, Cen Y, Zhu E, Li X, Chen D, Wang B. Cysteine-rich protein 61 regulates adipocyte differentiation from mesenchymal stem cells through mammalian target of rapamycin complex 1 and canonical Wnt signaling. FASEB J. 2018; 32:3096–107.
https://doi.org/10.1096/fj.201700830RR
PMID:29401606

25. Zarjou A, Jeney V, Arosio P, Poli M, Zavaczki E, Balla G, Balla J. Ferritin ferroxidase activity: a potent inhibitor of osteogenesis. J Bone Miner Res. 2010; 25:164–72.
https://doi.org/10.1359/jbmr.091002 PMID:19821764

26. Sikura KÉ, Potor L, Szerafin T, Zarjou A, Agarwal A, Arosio P, Poli M, Hendrik Z, Méhes G, Oros M, Posta N, Beke L, Fürtös I, et al. Potential Role of H-Ferritin in Mitigating Valvular Mineralization. Arterioscler Thromb Vasc Biol. 2019; 39:413–31.
https://doi.org/10.1161/ATVBAHA.118.312191
PMID:30700131

27. Peruzzi B, Cappariello A, Del Fattore A, Rucci N, De Benedetti F, Teti A. c-Src and IL-6 inhibit osteoblast differentiation and integrate IGFBP5 signalling. Nat Commun. 2012; 3:630.
https://doi.org/10.1038/ncomms1651
PMID:22252554
